# Integrating urban traffic models with coastal flood maps to quantify the resilience of traffic systems to episodic coastal flooding

**DOI:** 10.1016/j.mex.2021.101483

**Published:** 2021-08-09

**Authors:** Indraneel G. Kasmalkar, Katherine A. Serafin, Jenny Suckale

**Affiliations:** a Institute for Computational and Mathematical Engineering, Stanford University, Stanford, California, 94305 USA; b Department of Geography, University of Florida, Gainesville, Florida, 32611 USA; c Department of Geophysics, Stanford University, Stanford, California, 94305 USA; d Department of Civil and Environmental Engineering, Stanford University, Stanford, California, 94305 USA

**Keywords:** Coastal flooding, Transportation, Resilience, Sea level rise

## Abstract

Sea level rise and coastal floods are disrupting coastal communities across the world. The impacts of coastal floods are magnified by the disruption of critical urban systems such as transportation. The flood-related closure of low-lying coastal roads and highways can increase travel time delays and accident risk. However, quantifying the flood-related disruption of the urban traffic system presents challenges. Traffic systems are complex and highly dynamic, where congestion resulting from road closures may propagate rapidly from one area to another. Prior studies identify flood-related road closures by spatially overlaying coastal flood maps onto road network models, but simplifications within the representation of the road network with respect to the coastline or creeks may lead to an incorrect identification of flooded roads. We identify three corrections to reduce potential biases in the identification of flooded roads:

1. We correct for the geometry of highways;

2. We correct for the elevation of bridges and highway overpasses; and

3. We identify and account for road-creek crossings.

Accounting for these three corrections, we develop a methodology for accurately identifying flooded roads, improving our ability to quantify flood impacts on urban traffic systems and accident rates.

## Graphical Abstract

**Figure:**
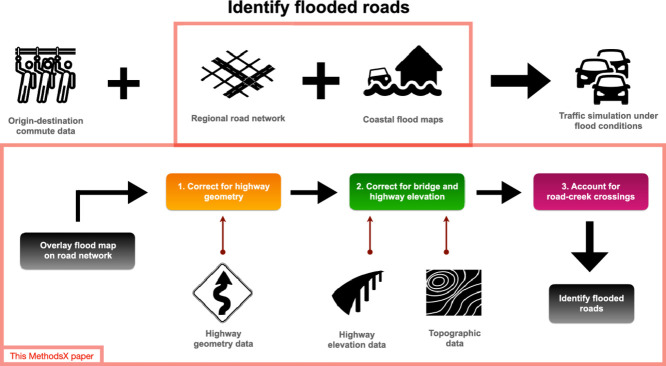

Specifications Table

**Table:** 

Subject Area:	Environmental Science
More specific subject area:	Urban traffic modeling
Method name:	Geospatial corrections to road networks for identifying flooded roads
Name and reference of original method:	P. Suarez, W. Anderson, V. Mahal, T. R. Lakshmanan, Impacts of flooding and climate change on urban transportation: A systemwide performance assessment of the Boston Metro Area. *Transportation Research Part D: Transport and Environment***, 10**, 231–244 (2005).
Resource availability:	http://cees-gitlab.stanford.edu/sigma/flood-related-traffic-disruption

## Method details

### Simplifications within the road network model may overestimate flooding on road segments

A road traffic assignment model populates road segments with commuters traveling from their specific origins to destinations. It requires two inputs: origin-destination commute demand data and a road network model. The first input, the origin-destination commute demand data, indicates the number of commuters who perform trips from a given origin to a given destination. Prior traffic studies based in the U.S. have used the Census Bureau's Longitudinal Employer Household Dynamics Origin Destination Employment Statistics (LODES) dataset [1] regarding locations of homes and workplaces at the census block level [2], [3], [4]. Since the LODES dataset does not incorporate information about the time or mode of commute, we augment the data with the American Community Survey [5] that provides estimates for the distribution of commuters by time and mode of commute.

The second input to the traffic assignment model, the road network model, represents the individual road segments and their intersections within the region, with additional data such as road capacity, number of lanes, and free flow travel speed. Prior simulation-based studies have performed simplifications to the road network models to reduce the computational complexity of traffic simulations [6]. Simplifications include aggregating local road segments into artificial roads, replacing curved road segments with straight segments, and removing data associated with vertical position, such as the elevation of bridges, highway overpasses, and tunnels. While the simplifications in road geometry and elevation cause minimal errors in stand-alone traffic simulations, they may introduce biases when estimating flood impacts [3].

The study by Suarez et al. [7], was the first to simulate traffic patterns under flood conditions by integrating a traffic assignment model for the Boston metro area, U.S.A., with riverine and coastal flood maps. The study spatially overlays flood maps on top of the regional road network model to identify potentially flooded road segments which are then closed down prior to the simulation. Subsequent studies have used the spatial overlay approach when quantifying flood impacts on the traffic system in other regions [2,^8^,9]. Suarez at al. highlight in a footnote the challenges of validating whether the spatial overlay approach accurately identifies flooded roads. They validated their results by conducting visual inspections of each potentially flooded road to verify the accuracy of the model's results. However, visual inspections might not be practically feasible for large study regions.

The study by Kasmalkar et al. [3] identified two sources of bias in the spatial overlay approach that may lead to an incorrect identification of flooded roads. First, the simplification of curved road segments into straight road segments may cause road segments to incorrectly intersect with areas of flooding. Second, the lack of elevation data for highway overpasses, bridges, as well as relatively small roads crossing over creeks may lead to spurious flooding on elevated road segments in the model.

Here, we provide the technical details for three corrections highlighted in Kasmalkar et al. [3] for reducing the biases in identifying flooded roads using the spatial overlay approach. The first correction addresses the simplification of curved road segments into straight line segments. The second correction addresses the lack of elevation data of large-scale road features such as highway overpasses and bridges. The third correction addresses the lack of elevation data for relatively smaller-scale roads crossing over creeks.

### Correction 1. Highway geometry

We define highways as numbered roads, e.g., US-101. To correct for the geometry of simplified highway segments, we first acquire a highway dataset with accurate geometries. For U.S.—based regions, the U.S. Census Bureau's Topologically Integrated Geographic Encoding and Referencing (TIGER) database [10] contains highway geometry data.

Each road segment within the road network model is flanked by two endpoints. As shown in Fig. 2A, for each highway segment in the road network model, we map the two end points to the respective nearest endpoints from the highway dataset with the same route number. We then identify the sequence of vertices and edges in the highway network between the two points. Identifying the sequence of vertices and edges may present a challenge if the segment within the highway dataset has multiple lanes. To remedy the problem, we pre-process the highway dataset so that only one lane from each highway is retained. To do so, we either use the Merge Divided Roads functionality of ArcGIS software, or if the lanes are numbered, we filter the highway dataset to keep only the first lane of each highway segment. We finally replace each simplified highway segment in our road network model with the corresponding sequence of vertices and edges identified within the highway dataset. With this correction, we alter the geometry and location of road segments within the road network model but we retain the underlying topological structure of the network.

**Fig. 1:**
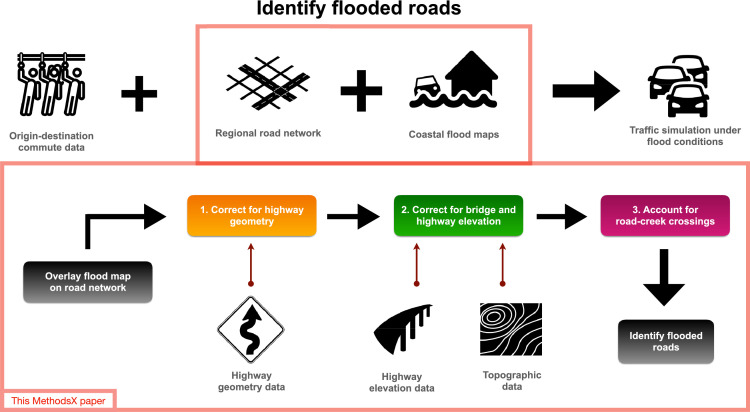
The model blueprint for quantifying flood-related traffic disruption. The three corrections to the methodology of identifying flooded roads are shown in the lower bounding box.

**Fig. 2:**
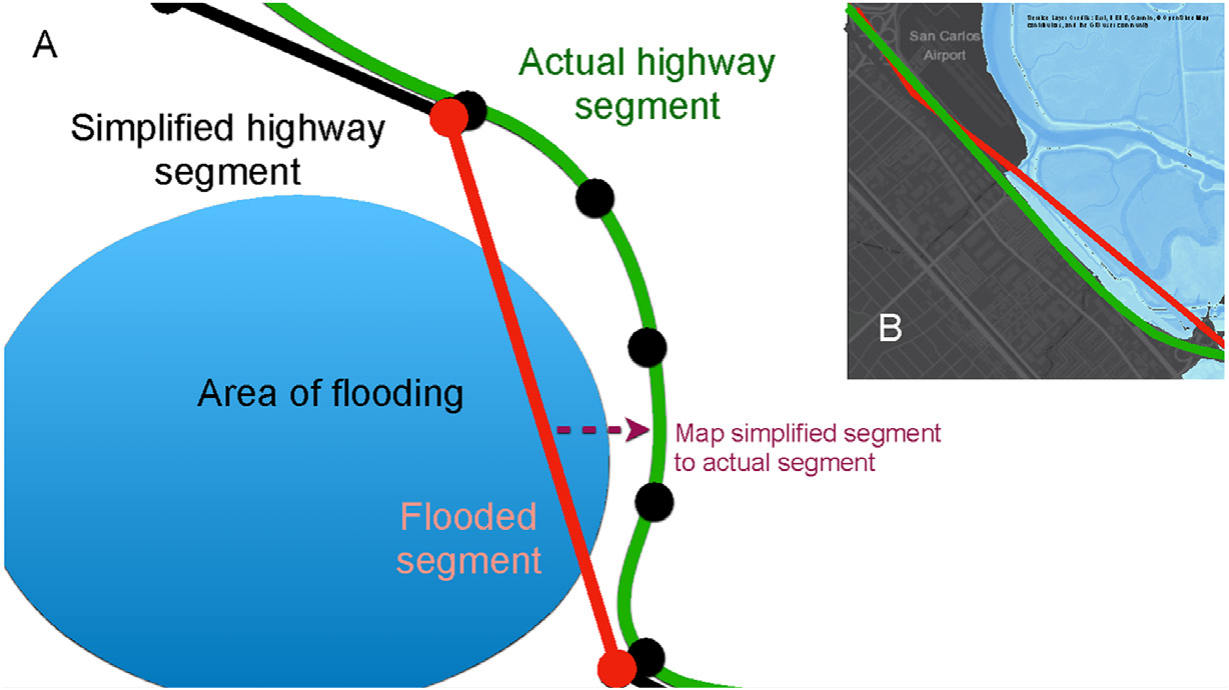
The highway geometry correction. A: Schematic representation. The simplified highway segment is flooded (shown in red) because its straight-line geometry incorrectly intersects the area of flooding (shown in blue). The actual highway segment (shown in green) curves around the area of flooding. The correction maps the simplified highway segment to the actual segment (shown in dashed arrows). B: Example of geometry correction for the US-101, California. Panel B is derived from Fig. 2A of Kasmalkar, I.G., Serafin, K.A., Miao, Y., Bick, I.A., Ortolano, L., Ouyang, D., Suckale, J., 2020. When floods hit the road: Resilience to flood-related traffic disruption in the San Francisco Bay Area and beyond. *Science Advances* 6 32, eaba2423, distributed under a Creative Commons Attribution Non-Commercial License 4.0 (CC BY-NC) http://creativecommons.org/licenses/by-nc/4.0/ .

In Fig. 2B, we show the example of a segment of the US-101 near San Carlos, California, which curves around the area of flooding but whose simplified version intersects the area of flooding. The highway geometry correction remedies the error for the given example. We perform the correction for each highway segment in the region. The correction is not used for non-highway segments because these segments typically represent aggregated versions of local roads and thus may not correspond to real road segments.

### Correction 2. Bridge and highway elevation

We incorporate elevation data to correct for the elevation of bridges and highway overpasses. We define bridges as numbered roads segments greater than 2000 ft in length and running over large stretches of water bodies, such as the Golden Gate Bridge. Elevation data for bridges and highway overpasses for some regions may be obtained from regional transport authorities. The study by Kasmalkar et al. [3] uses accelerometer-based elevation data for the San Francisco Bay Area collected by the California Department of Transportation [11].

**Fig. 3:**
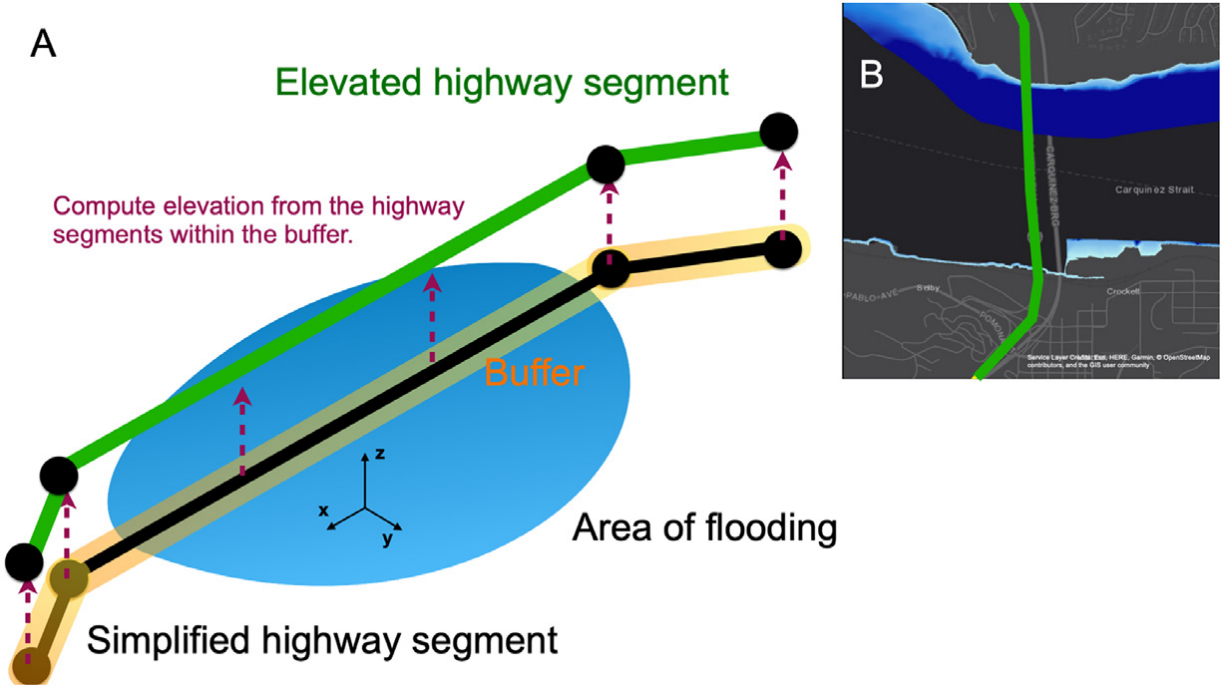
The elevation correction. A: Schematic representation. The simplified highway segment (shown in black) is buffered (shown in yellow) so that the corresponding actual highway segment (shown in green) can be easily identified by planar intersection with the buffer. The elevation data of the actual highway segment is then mapped to the simplified highway segment. B: Example of elevation correction for the Carquinez Bridge, California. Panel B is derived from Fig. 2B of Kasmalkar, I.G., Serafin, K.A., Miao, Y., Bick, I.A., Ortolano, L., Ouyang, D., Suckale, J., 2020. When floods hit the road: Resilience to flood-related traffic disruption in the San Francisco Bay Area and beyond. *Science Advances* 6 32, eaba2423, distributed under a Creative Commons Attribution Non-Commercial License 4.0 (CC BY-NC) http://creativecommons.org/licenses/by-nc/4.0/ .

We show the procedure for joining highway elevation data to the highway segments of the simplified road network model in Fig. 3A. First, we discard all the non-highway road segments from the simplified road network model. We then use the ArcGIS Buffer tool to create a 100–200 ft width band around each highway segment. The buffer facilitates the spatial join of the highway road segments from the simplified road network model to those from the highway elevation dataset. If the highway geometry correction is applied prior to performing the highway elevation correction, then the use of the buffer may be avoided since the highway segments will align with those in the highway elevation data. However, the buffer approach makes the spatial join procedure robust to small errors in the geometry.

After buffering, we use the ArcGIS Spatial Join tool to map segments from the highway elevation dataset onto the buffered highway segments. For each buffered highway segment, we average the elevation value over all segments from the highway elevation dataset that intersect the buffer. We note that this procedure may introduce errors at the intersections of highways, since segments from other highways may also fall within the buffer near the intersection. To reduce the error, we use a conditional spatial join that only considers highway segments with a given route number.

To compare the elevation of the highways against the flood depth, we transform both pieces of information to a common baseline. Each pixel within the flood map represents the depth of the water, which is independent of any vertical datum. On the other hand, elevation data in the United States is generally presented relative to vertical datums such as the World Geodetic System 1984 vertical datum, or the North American Vertical Datum of 1988.

To remedy the discrepancy in vertical datum, we convert the highway elevation values into datum-independent above-ground elevation values. We acquire a Digital Elevation Model (DEM), namely, a topographic map, to compare the highway elevation to the elevation of the ground. The United States Geological Survey provides high resolution DEM raster maps for most areas of the country [12,13]. We first project both the topographic and highway elevation data onto the same vertical datum. Then, for each highway segment, we identify all the intersecting DEM pixels and record the average topographic elevation value of the pixels. Finally, we subtract the average topographic elevation from the computed highway elevation to obtain the above-ground elevation of the highway segment.

In Fig. 2B, we show the example of the Carquinez Bridge, California, which is elevated approximately 400 ft above the Carquinez Strait, but is incorrectly deemed as flooded in the absence of elevation data. The elevation correction remedies the error. We apply the elevation correction to all highway segments within the given region. Similar to the case of geometry correction, the elevation correction method is not applied to non-highway road segments within the road network model since such segments may be artificial or aggregated versions of multiple road segments.

### Correction 3. Identifying and accounting for road-creek crossings

**Fig. 4:**
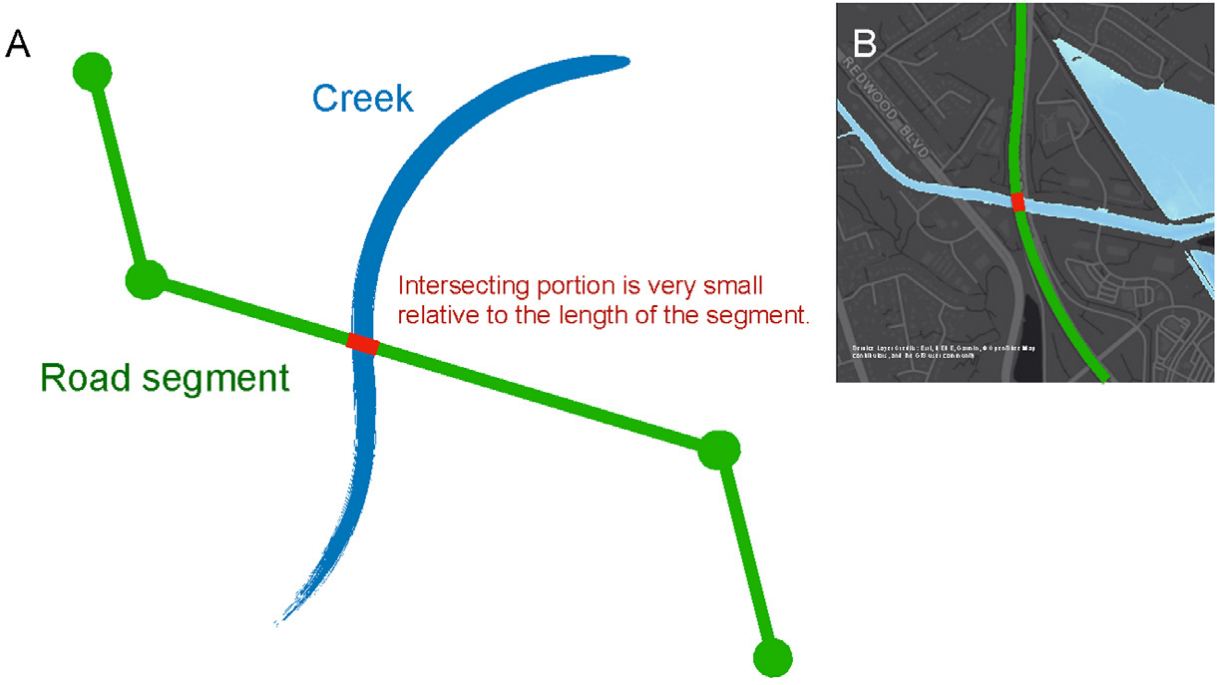
Identifying and accounting for road-creek crossings. A: Schematic representation. The simplified road segment (shown in green) intersects the creek (shown in blue) over a very small length (shown in red). B: Example of the US-101 crossing the San Rafael creek, California. Panel B is derived from Fig. 2C of Kasmalkar, I.G., Serafin, K.A., Miao, Y., Bick, I.A., Ortolano, L., Ouyang, D., Suckale, J., 2020. Supplementary Materials for When floods hit the road: Resilience to flood-related traffic disruption in the San Francisco Bay Area and beyond. *Science Advances* 6 32, eaba2423, distributed under a Creative Commons Attribution Non-Commercial License 4.0 (CC BY-NC) http://creativecommons.org/licenses/by-nc/4.0/ .

Although the elevation correction corrects flooding issues for bridges and highway overpasses crossing over large water bodies such as the San Francisco Bay, there are still issues with flood estimation for roads running over narrower creeks. We define creeks as water bodies whose width is no more than 200 ft. Elevated coastal water levels cause water to flow up rivers and creeks, potentially exposing roads passing over rivers and creeks to flooding. During flood map development, road segments that cross creeks are often removed from the underlying DEM to facilitate continuity in water flow upstream of the road-creek crossings. Thus, when overlaying the flood map on the road network, small segments of roadways appear to be flooded when in reality, the road would cross over the creek/river (Fig. 4B).

The elevation correction does not remedy the false instances of flooding for road-creek crossings because of the relatively smaller length scales of creeks. At these smaller scales, the topographic elevation of the creek bed within the DEM is approximately similar to that of the areas surrounding the creek, thus introducing errors in estimating the above-ground elevation of road segments. Moreover, the elevation correction only applies to highway segments, since elevation data may not be available for all non-highway segments. Although individual non-highway segments have relatively small traffic volumes, the systematic false flooding of all non-highway segments crossing a particular creek may accumulate into a substantial error in the estimation of regional traffic flow. Therefore, we present a third correction to identify and account for false instances of flooding at road-creek crossings.

As shown in Fig. 4A, the key insight of the correction is that, given the relatively narrow widths of creeks, road segments that cross a creek have only a very small extent that intersects the creek. Thus, for each road segment, we analyze the percentage of road segment length that is flooded for a given water level. Since the flood maps are represented in raster format, we quantify the percentage of flooded road segment length by calculating the number of flooded pixels intersecting the road segment divided by the number of pixels over the length of the entire road segment.

**Fig. 5:**
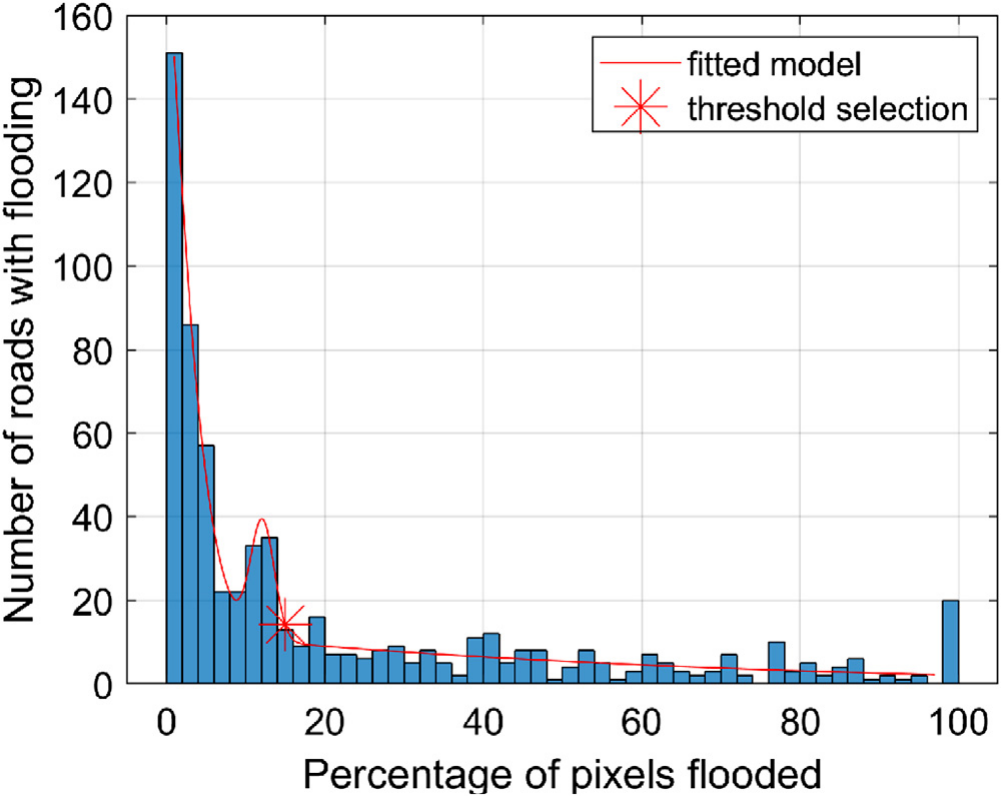
The histogram of the percentage of segment length flooded for road segments in the San Francisco Bay Area, U.S.A., for the 12 in. coastal water level. The segment length is measured in terms of the number of pixels of the flood map intersecting with the segment. The figure is reprinted from Kasmalkar, I.G., Serafin, K.A., Miao, Y., Bick, I.A., Ortolano, L., Ouyang, D., Suckale, J., 2020. Supplementary Materials for When floods hit the road: Resilience to flood-related traffic disruption in the San Francisco Bay Area and beyond. *Science Advances* 6 32, eaba2423, distributed under a Creative Commons Attribution Non-Commercial License 4.0 (CC BY-NC) http://creativecommons.org/licenses/by-nc/4.0/ .

The histogram by Kasmalkar et al. [3], shown in Fig. 5, summarizes the distribution of the percentages of flooded road segment lengths for the San Francisco Bay Area for 12 in. of flooding, with three peaks in the histogram. The left-most peak is at 0%, indicating that the majority of road segments around the bay are not flooded. The right-most peak is at 100%, indicating completely flooded road segments. The peak in the middle indicates a relatively large number of road segments experiencing between 14–20% of the roadway flooded, suggesting that these road segments overlap with relatively narrow water bodies such as creeks. To identify flooding generated by road-creek crossings, we impose a threshold related to the percentage of flooded road segment length below which we deem that a road is not flooded. To identify a threshold, we fit a Gaussian model to the histogram for each coastal water level considered within the study and set the threshold as the inflection point between the middle and right peaks of the distribution. The averaged threshold across all coastal water levels is estimated to be 17% for the San Francisco Bay Area [3]. In other words, only those road segments which are more than 17% inundated are considered as flooded in the study by Kasmalkar et al. [3].

### Quantifying traffic disruption under flood conditions

After applying all three corrections to identify flooded roads, we follow Kasmalkar et al. [3] and assume that flooded road segments with more than three inches of water at any point across their extent are closed down to traffic flow. For flooded road segments with less than three inches of water, the study by Pregnolato et al. [14] suggests a function based on water level for reducing the free flow travel speed for a flooded road segment.

After the roads are closed, the traffic assignment model simulates regional traffic flow under flood conditions. We then compare the traffic volumes and travel times in the presence and absence of flooding to quantify the flood-related disruption of traffic patterns. The study by Kasmalkar et al. [3] investigates the factors that govern flood-related travel time delays, such as the availability of alternate roads. The study by Kasmalkar and Suckale [4] use regression-based methods to combine the simulated traffic volume with historical accident data for cars and pedestrians, and then use the regressions to predict the accident rates for communities in the San Francisco Bay Area under flood conditions.

### Error analysis of the three proposed corrections for the San Francisco Bay Area

**Fig. 6:**
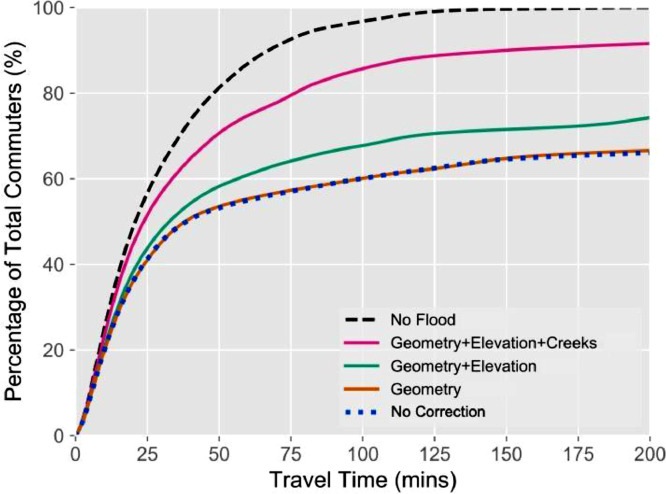
Cumulative distribution of travel time for commuters across the San Francisco Bay Area under flood conditions and for the three corrections. The figure is derived from Kasmalkar, I.G., Serafin, K.A., Miao, Y., Bick, I.A., Ortolano, L., Ouyang, D., Suckale, J., 2020. When floods hit the road: Resilience to flood-related traffic disruption in the San Francisco Bay Area and beyond. *Science Advances* 6 32, eaba2423, distributed under a Creative Commons Attribution Non-Commercial License 4.0 (CC BY-NC) http://creativecommons.org/licenses/by-nc/4.0/ .

Using results from Kasmalkar et al. [3], we present an error analysis for our methodology of identifying flooded roads for the San Francisco Bay Area, California. The datasets used for modeling morning commute patterns in the presence of flooding are summarized in Table 1. In Fig. 6, derived from Kasmalkar et al., we highlight the cumulative distributions of estimated commuter travel times for the three corrections. We also present the distribution of commuter travel times for the no-flood condition, shown as a dashed line. We quantify flood-related traffic disruption as the increase in travel time under flooding conditions over the baseline no-flood condition.

**Table 1:** Datasets used in the study by Kasmalkar et al. [3] to quantify flood-related traffic disruption in the San Francisco Bay Area, California.

Dataset template	Specific dataset	Description
Regional road network	San Francisco Bay Area Metropolitan Transportation Commission Road Network [15].	A simplified regional road network that contains all the highways and aggregated versions of road segments.
Coastal flood map	San Francisco Bay Area Adapting to Rising Tides flood maps [16].	Coastal flood maps depicting water depths for the 12-, 24- and 36-inch coastal water levels in the region.
Origin-destination commute data	Longitudinal Employer Household Dynamics Origin Destination Employment Statistics (LODES) dataset [1].	A dataset prepared by the United States Census Bureau that provides home and workplace census blocks for commuters per state.
Highway map	California TIGER/Line Primary and Secondary roads [10].	A geodatabase that provides the primary and secondary roads in the state of California with accurate geometries.
Highway elevation data	California Department of Transportation highway elevation dataset [11].	A geodatabase that provides GPS—based elevation data for highways in the state of California.
Topographic data	United States Geological Survey 1-m resolution bathymetric map [12].	Topographic maps for the North-west American region.
Commute survey	American Community Survey 2013-2017 [5].	The American Community Survey provides the proportion of commuters leaving for work at a given time and using a particular mode of commute.

We first present the distribution of travel times for the 36 in. coastal water level in the absence of corrections, shown as a dotted line. While 80% of commuters reach their workplaces within 50 min in the absence of flooding, only 53% of commuters reach their workplaces in the same amount of time in the presence of flooding. However, the traffic model overestimates the impact of flooding because of the lack of road geometry and elevation data. We perform the corrections described in this study and highlight the corresponding changes in the distributions of travel times.

The geometry correction, shown in orange, causes a relatively small change in travel times over the absence of corrections, a result of the internal cancellations between travel time increases on some corrected highways and decreases on others. For example, some highway segments, such as the US-101 in Fig. 2B, curve around the areas of flooding so that the geometry correction reduces estimated travel times for commuters. In other cases, the geometry correction may modify simplified segments so that they intersect the areas of flooding, increasing travel times.

The elevation correction, shown in green, causes a notable reduction in travel times. The San Francisco Bay Area has several bridges which act as key nexus traffic points, such as the Golden Gate Bridge and the Bay Bridge, that facilitate regional traffic flows across the San Francisco Bay [3]. In the absence of the elevation correction, the flooding of these bridges greatly overestimates travel time for commuters.

The correction for road-creek crossings, shown in magenta, accounts for the largest reduction in travel time. In Fig. 6, we see that the percentage of commuters who reach their workplaces within 50 min increases from 53% to 60% when the geometry and elevation corrections are applied. With the correction for road-creek crossings, the number increases further to 70%. Even though road-creek crossings are relatively small-scale features compared to bridges and highway overpasses, our results suggest that the cumulative effect of overestimating flooding across multiple road-creek crossings may cause substantial errors in modeling regional traffic flows.

Overall, the combined effect of the three corrections indicates a substantial reduction in the overestimation of flood impacts on the urban traffic system in the San Francisco Bay Area, highlighting the importance of these corrections when integrating traffic models with flood maps.

### Automatization of the method

To make our method automatable, we provide Python-based code (see Resource Availability) that overlays a given road network on top of a set of flood maps, incorporates geometry and elevation data, and applies the three proposed corrections. The Python code takes as input a shapefile for the road network. To apply the geometry and elevation corrections, the code requires that each road segment be classified with either the highway route number or as a ‘non-highway’ road. The code also takes pixel-based raster files for flood maps, where the color of each pixel corresponds to the height of the water above the ground. For the corrections, the code also takes in additional shapefiles with road network geometry and elevation. The run-time of our code to identify flooded road segments in the San Francisco Bay Area, for which our road network has approximately 33,000 road segments, is less than 10 min. Since different regions may have different data types and conventions, the code may not be directly applicable to datasets from other regions and agencies but provides a base script which can be modified to match other data types.

## The need for continued validation

The goal of our method is to identify road segments that would flood under a range of extreme water levels. Validating the method is an ongoing challenge because there is limited empirical data about the road segments that have flooded in the past. The low water levels considered here fall into the category of so-called ‘nuisance flooding’ - relatively common, low-grade flooding - that leads to water levels that are not necessarily extreme enough to merit detailed documentation. Recently, citizen science projects like the California King Tides Project by the California Coastal Commission have made progress towards the goal of recording locations of low-grade flooding more systematically, but do not yet provide a comprehensive enough dataset to enable systematic validation [17]. Some of the relatively higher water levels considered in our study, such as the 36 inch water level, have not occurred in the past and are hence unobserved.

Flood models such as those from the Adapting to Rising Tides program [16] attempt to fill the gap between past, observed conditions and future, uncertain conditions. However, detailed flood projections in an inevitably dynamic urban system are very challenging. Small-scale interventions, such as temporary flood blockages through sandbags or clogged drains, can alter the water depth on individual roads significantly. Even the Digital Elevation Models on which flood maps are based have uncertainties of up to a meter, particularly in the South Bay where salt marshes line the inner bay. Finally, it is important to keep in mind that many of the smaller roads in the model are an aggregate representation of the road network and rather than corresponding to one specific, physical road [6].

These challenges raise the question of what information the model can provide in its current form and how to validate it. We emphasize that the improvements we suggest here provide a starting point for identifying flooded roads more accurately. However, continued validation via multiple sources is required. One potential form of validation involves using social media data, such as photos of flooded roads [17], to identify flood exposure. Aerial imagery during flood events may provide additional means of large-scale validation. For the San Francisco Bay Area in particular, a prior study validates modeled changes in accident rates caused by flooded roads against historical accident data during flood conditions, providing an indirect means of validation of the method of identifying flooded roads [14].

## Conclusions and limitations

Urban traffic systems in coastal regions are facing increasing risks of disruption from a rising global sea level and intensifying coastal storms. To quantify the flood-related disruption of traffic, prior studies have identified potentially flooded roads by spatially overlaying flood maps on top of the regional road network. However, integrating models designed for distinct purposes, such as road networks and flood maps, may introduce notable biases. For example, simplifications within the road network model, intended for computational efficiency, may cause an overestimation of flooding on certain segments. Our study presents three corrections to the spatial overlay approach to reduce the overestimation of flood impacts on the road network. First, we correct the geometry of highway segments to reduce the false intersection of road segments with the areas of flooding. Second, we correct for the elevation of bridges and highways overpasses so that the model does not incorrectly deem certain segments as flooded. Third, we identify road segments crossing creeks and impose a threshold related to the percentage of flooded road length to remove false instances of flooding. The three corrections provide a more accurate identification of potentially flooded roads, and thus improve the quantification of flood impacts on the urban traffic system.

Even with the use of the three corrections, we emphasize that there are multiple other sources of error that limit the validity and applicability of model-based approaches for quantifying flood-related traffic disruption. Sources of error include the uncertainties in the mapping of coastal flood water levels, and simplifications within the road network designed for computational efficiency. Traffic volumes and travel times derived from models are representative of typical conditions, and do not incorporate adverse road conditions such as construction work, closed lanes, or obstacles, which can notably impact traffic conditions. Uncertainty in urban growth and the adoption of new technologies such as autonomous vehicles and remote work can also substantially alter near-future commute patterns and introduce errors into the estimates of flood-related traffic disruption.

## Resource availability

The Python code for identifying flooded roads and applying the three corrections highlighted in this paper is available at http://cees-gitlab.stanford.edu/sigma/flood-related-traffic-disruption.

## Declaration of Competing Interest

The authors declare that they have no known competing financial interests or personal relationships that could have appeared to influence the work reported in this paper.
